# Visualization of the internal structure of *Didymosphenia geminata* frustules using nano X-ray tomography

**DOI:** 10.1038/s41598-017-08960-5

**Published:** 2017-08-22

**Authors:** Izabela Zgłobicka, Qiong Li, Jürgen Gluch, Magdalena Płocińska, Teresa Noga, Romuald Dobosz, Robert Szoszkiewicz, Andrzej Witkowski, Ehrenfried Zschech, Krzysztof J. Kurzydłowski

**Affiliations:** 1 Faculty of Materials Science and Engineering, Warsaw University of Technology, 141 Wołoska Str., 02–507 Warsaw, Poland; 2 Fraunhofer–Institut für Keramische Technologien und Systeme IKTS, Maria–Reiche–Strasse 2, 01109 Dresden, Germany; 3 Dresden Center for Nanoanalysis, Technische Universität Dresden, 10 Helmholtzstraße, 01069 Dresden, Germany; 4 Faculty of Biology and Agriculture, University of Rzeszów, 1 Ćwiklińskiej Str., 35–601 Rzeszów, Poland; 5 Faculty of Chemistry, University of Warsaw, 1 Pasteura Str., 02–093 Warsaw, Poland; 6 Faculty of Geosciences, Paleoceanology Unit, Natural Science Research and Educational Center, University of Szczecin, 18 Mickiewicza Str., 70–383 Szczecin, Poland; 7 Faculty of Mechanical Engineering, Bialystok University of Technology, 45C Wiejska Str., 15–351 Bialystok, Poland

## Abstract

For the first time, the three-dimensional (3D) internal structure of naturally produced *Didymosphenia geminata* frustules were nondestructively visualized at sub-100 nm resolution. The well-optimized hierarchical structures of these natural organisms provide insight that is needed to design novel, environmentally friendly functional materials. Diatoms, which are widely distributed in freshwater, seawater and wet soils, are well known for their intricate, siliceous cell walls called ‘frustules’. Each type of diatom has a specific morphology with various pores, ribs, minute spines, marginal ridges and elevations. In this paper, the visualization is performed using nondestructive nano X-ray computed tomography (nano-XCT). Arbitrary cross-sections through the frustules, which can be extracted from the nano-XCT 3D data set for each direction, are validated via the destructive focused ion beam (FIB) cross-sectioning of regions of interest (ROIs) and subsequent observation by scanning electron microscopy (SEM). These 3D data are essential for understanding the functionality and potential applications of diatom cells.

## Introduction

Diatoms (Bacillarophyceae) are unicellular, eukaryotic and photoautotrophic organisms that inhabit aquatic and terrestrial environments. An estimate of diatom diversity ranges from 100,000 to 200,000 species^1–3^. Diatoms are characterized by the presence of a siliceous cell wall called a ‘frustule’. The size of a single diatom frustule ranges between 1 µm and 5.6 mm^4–6^. The species from the lowermost region of the spectrum can range from 1 µm to a couple of dozen micrometres (e.g. Li *et al*., 2016)^7^. Likewise, the species from the largest part of the size spectrum can exceed 5 mm at the maximum but are never smaller than ca. 0.8–1.0 mm. Despite a broad size range of the siliceous exoskeleton in diatom cells, their ultrastructural elements (ornamentation) possess a strict pattern and quantitative measure. Morphometric characters, where characters are described in proportion to the cell size, such as the density of ribs, stria, or pores in 10 µm, or mathematical descriptions of the pore shape have become more common in diatom identification. These morphometric characters have been shown to be species specific and genetically controlled^8^. Therefore, if the measurements are performed on cells across the size range, these patterns of ornamentation remain the same. For example, the stria density in 10 µm will remain within the same range in a species, regardless of cell size.

The diatom frustule is mainly composed of biogenic opaline silica and either organic polymers^9^ or chitin^10^. The frustule comprises two overlapping valves with a few to many girdle bands that enclose the protoplast^11–15^. On the basis of the frustule symmetry, diatoms are divided into two major groups: centrics (with either radial or bilateral symmetry) and pennates (with bilateral symmetry)^6, 12, 13^.

The diatom frustule, because of its overall shape and the presence of specific internal substructures (e.g. linking spines, long processes and setae), provides a protective role against grazers and predators^16^. Measurements of the elastic modulus and hardness of silica using nanoindentation have shown a variation in these material parameters that depends upon the location within the frustule^17, 18^. Likewise, the size of the diatom frustule influences its fracture resistance. Generally, a smaller cell size requires greater mechanical strength to break the frustule^19^. These mechanical properties appear to be the result of the frustule’s hierarchical architecture, particularly the presence of ribs or pores/areoles, which can dissipate the mechanical stress to the entire frustule. These particular mechanical properties originate from the unique and intricate microstructural and nanostructural design of the frustule^17, 20–22^.

The growing interest in studies of the siliceous component of diatoms has been stimulated by a large variety of applications ranging from those that take advantage of mechanical properties^19^ to medicine^23, 24^, electronics^25^ and biomimetics^6, 26^. These applications require knowledge of the internal structure of diatoms, which can be obtained via nondestructive high-resolution 3D imaging, so that the natural size, orientation and proximity of the cell components can be determined. Both nano X–ray tomography and focused ion beam (FIB)-based serial cutting techniques with subsequent imaging by scanning electron microscopy (SEM) are options to provide 3D information about the internal structure of diatom frustules. The main difference between these two techniques is that FIB–SEM is a destructive technique, whereas nano-XCT is a nondestructive technique. Furthermore, the slicing direction with FIB–SEM is limited, which causes a lack of options with regard to intersecting the entire diatom frustule (along the long axis). Problems with FIB–SEM reconstructions have been encountered with porous materials (like diatoms) because of excessive brightness and the so-called edge effect of SEM^27^, where electrons are more easily excited from the edge of a solid feature. The solution to this problem is pore impregnation with epoxy. The impregnation material must be properly matched with chemical composition, pore size, and fragility of the tested material. With nano-XCT, however, the contrast is achieved without additional preparation processes. An important fact is that FIB–SEM must be conducted in vacuum, whereas nano-XCT, which is conducted at multi-keV photon energies, does not require vacuum conditions.

This paper presents a novel approach for the high-resolution imaging of internal structures of diatom frustules via nano X-ray computed tomography (nano-XCT) complemented by SEM imaging of FIB cross-sections through the ROI, to nondestructively study diatoms and their biomineralization kinetics.

## Results and Discussion

**Figure 1:**
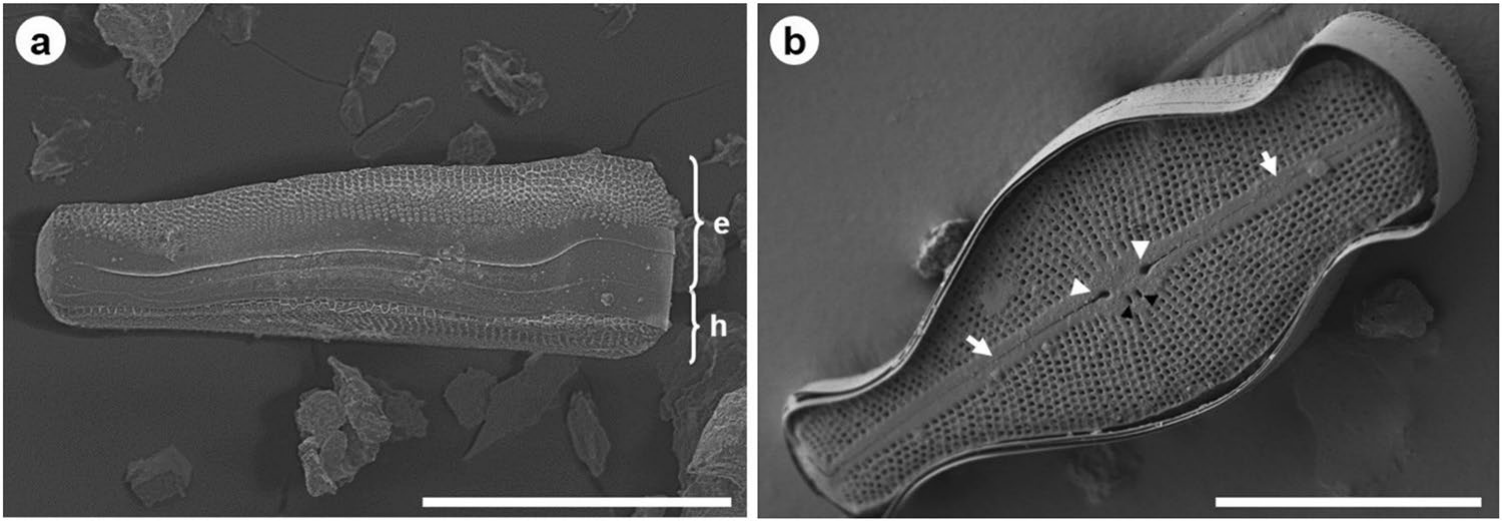
SEM images of *D. geminata* frustule: (**a**) girdle view (e – epivalve, h – hypovalve); (**b**) valve view. Note the presence of the apically oriented slit (*arrows*). The raphe was composed of two branches and two expanded pores. The external central raphe endings mark the extent of the central nodule (*white arrowheads*). The two closely located pores represent an opening of the stigmata (*black arrowheads*). Scale bars: 40 µm (**a**,**b**).

**Figure 2:**
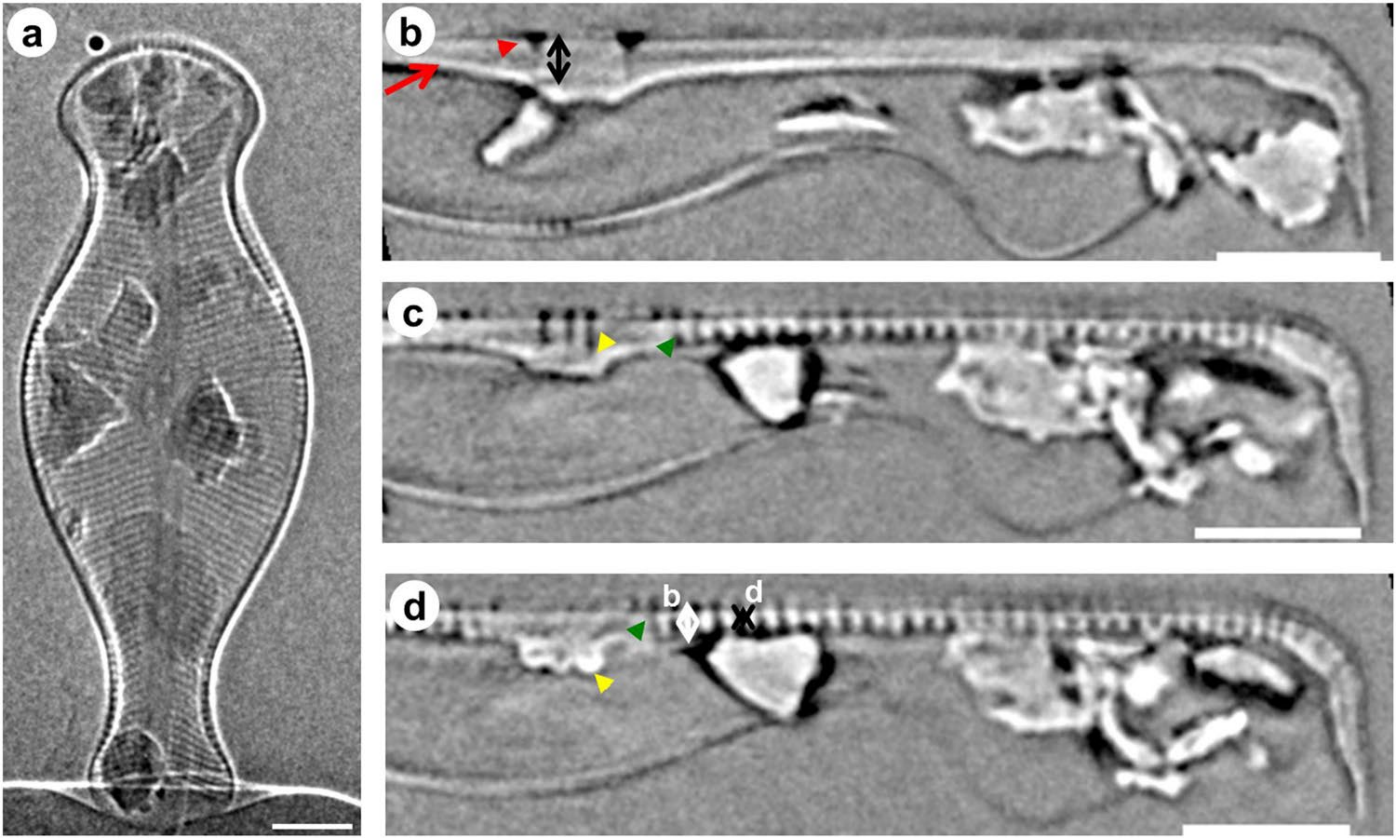
Nano–XCT results of the frustule of *D. geminata*. (**a**) One projection of the entire frustule of *D. geminata* imaged by nano–XCT in phase contrast mode. (**b**–**d**) One slice extracted from the reconstructed 3D volume in girdle view of the frustule. *Red arrowheads*: raphe, *red arrow*: raphe extending, *green arrowheads*: ribs, *yellow arrowheads*: stigmata, *white and double arrows*: width, height, and distance of the ribs and struts. Scale bars: 10 µm (**a**–**d**).

The visualization of the diatom frustule was conducted on *Didymosphenia geminata*, a biraphid pennate diatom. SEM images (Fig. 1) illustrate girdle (A) and valve (B) views of the *D. geminata* frustule. The images clearly show that one of the valves, the epivalve (Fig. 1a, marked with ‘e’) is larger than the other one, the hypovalve (Fig. 1a, marked with ‘h’). Nano-XCT studies of the frustules of *D. geminata* show both surface and internal structures of the frustules in a nondestructive manner based on a single tomographic data set. Figure 2a shows the entire frustule of *D. geminata* in one projection (exposure time of 180 s) as an arbitrary cross-section. From the 3D tomographic imaging, the radiograph shows all surface and internal structures; more details are distinguishable in individual cross-sections based on nano-XCT. Both X-ray and SEM images provide information regarding the geometry of the *D. geminata* frustule.

**Figure 3:**
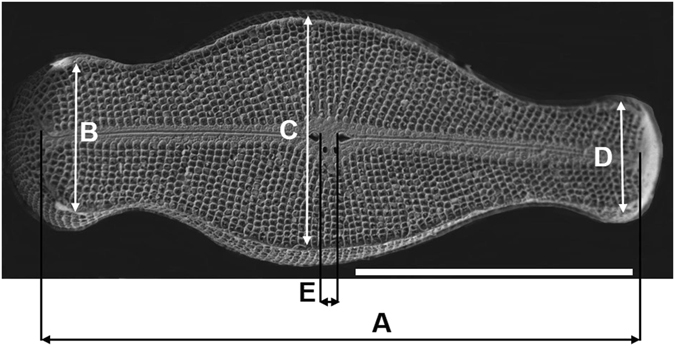
SEM image of the *D. geminata* frustule with marked size dimensions: *A* – apical axis of the frustule; *B* through *D* – transapical axis. Scale bar: 40 µm.

Measurements of the *D. geminata* frustule (including the length of the entire frustule; the greatest thickness at the head pole, central region and foot pole; and the distance between stigmata) are indicated in Fig. 3 and listed in Table 1. On the basis of these results, the frustule shows considerable asymmetry along the apical axis, which is expressed by the variations in the width of the frustule ends (apices) and the position of the centre of gravity.

**Table 1:** The mean values with standard deviations of *D. geminata* size dimensions (according to the designation in Figs 2–4).

Imaging method	Length (A) [µm]	Width, head pole (B) [µm]	Width, middle part (C) [µm]	Width, foot pole (D) [µm]	Distance between proximal ends (E) [µm]
SEM	86.7 ± 8.2	19.8 ± 2.1	30.9 ± 3.1	13.6 ± 1.6	4.9 ± 0.7
Nano–XCT	89.1	24.7	35.8	18.1	4.5

According to Dawson (1973) and Moffat (1994), *D. geminata* valves are relatively large compared with those of other diatoms; the valves are 120–140 µm in length and 35–45 µm in width^28, 29^. However, Spaulding (2010) claims a much larger size range for the valves of *D. geminata*: from 65 µm to 161 µm in length and from 36 µm to 41 µm in width^30^. On the basis of SEM images (n = 24) of the frustules, we calculated an average length (A) and width (C) of 87 ± 8 µm (A) and 31 ± 3 µm (C), respectively, for a single cell of *D. geminata*. These results suggest that these data originating from this study are smaller than the literature data. Specifically, our results show that the apical end (B) is approximately 1.5 times wider than the distal end (D) (Table 1).

**Figure 4:**
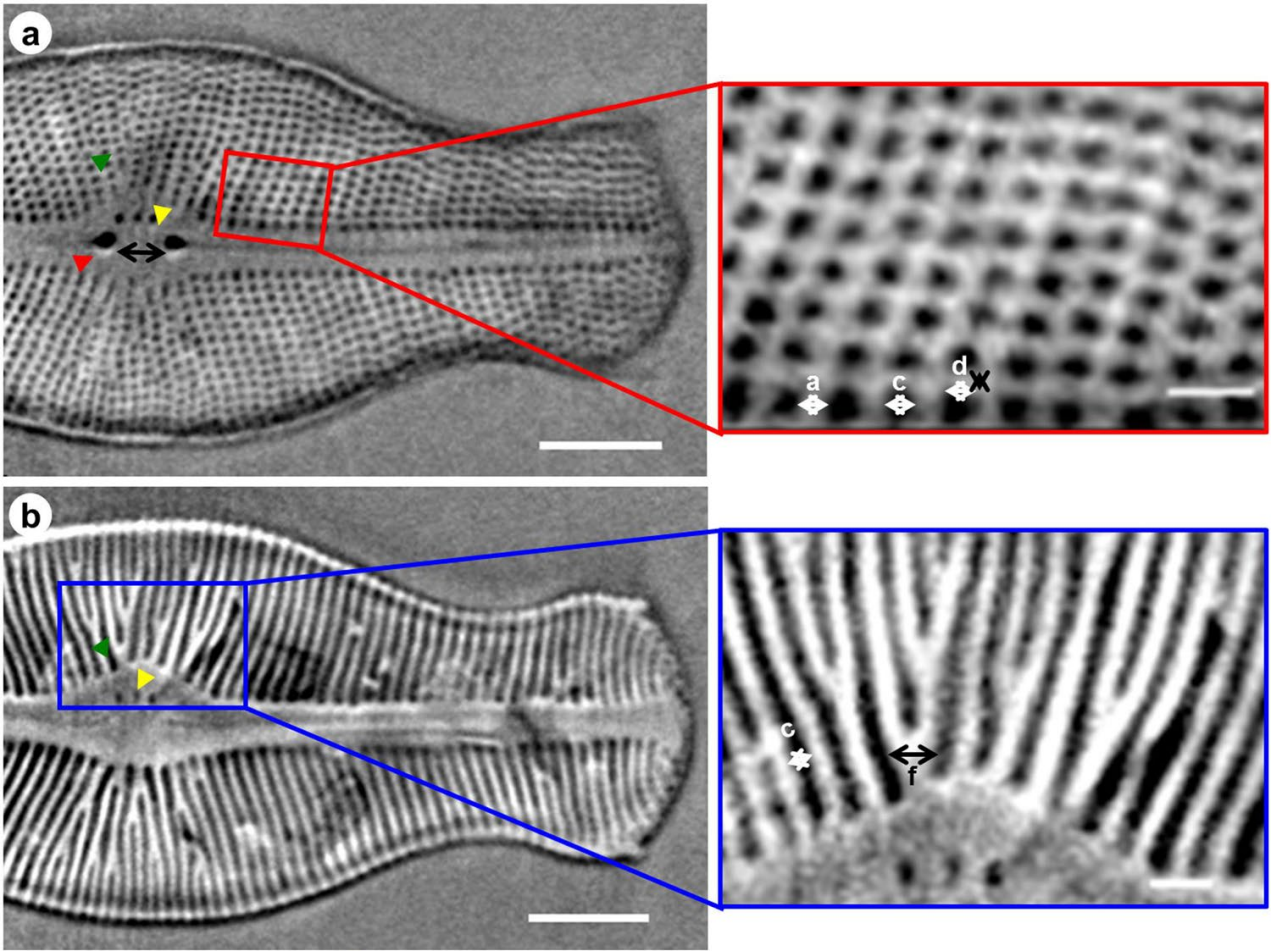
(**a**,**b**) Nano-XCT results of the valve view of the frustule of *D. geminata* from the surface to the valve interior in the Z direction (one slice extracted from the reconstructed 3D volume). Insets: zoomed regions of (**a**) (*red rectangle*) and (**b**) (*blue rectangle*). *Red arrowheads*: raphe, *green arrowheads*: ribs, *yellow arrowheads*: stigmas, *white* and *double arrows*: width, height, and distance of the ribs and struts. Scale bars: 10 µm (**a**,**b**); 2 µm (insets).

Each valve of *D. geminata* has two raphe branches that are both slits through the valve. According to Moffat (1994) and Aboal *et al*. (2012), in the central region of the valve, 3–6 stigmata are observed and are placed unilaterally near the external proximal ends of the raphe^29, 31^. Notably, the nano-XCT and SEM data (see Table 1E) show that the distance between the two central external raphe endings (red arrowhead in Fig. 4a) in this frustule ranges from 4.2 to 5.6 µm. Additionally, the depth of the openings of the external central raphe endings was measured (black double arrow in Fig. 2b) and shown to be approximately 3.6 µm (Table 2A). Each external proximal raphe end forms a cone in the centre of the frustule and extends towards the apices. During our investigations, the frustules of *D. geminata* possessed two or three stigmata (yellow arrowheads in Figs 2c,d and 4b,c), which are localized near the proximal raphe ending with a width of 0.7 µm and a depth of 1.6 µm (Table 2B and C). Stigmata appear as isolated pores (Fig. 4a,b) in the valve view and open internally with a complex morphology that is resolved in the girdle view (Fig. 2c,d).

**Table 2:** Average values with standard deviations of other parameters of the *D. geminata* cell structure from the nano-XCT tomography data set (marked in Figs 2 and 4).

Imaging method	Depth of central raphe ending (A) [µm]	Width of stigma (B) [µm]	Depth of stigma (C) [µm]
Nano-XCT	3.58 ± 0.05	0.69 ± 0.065	1.59 ± 0.26

**Figure 5:**
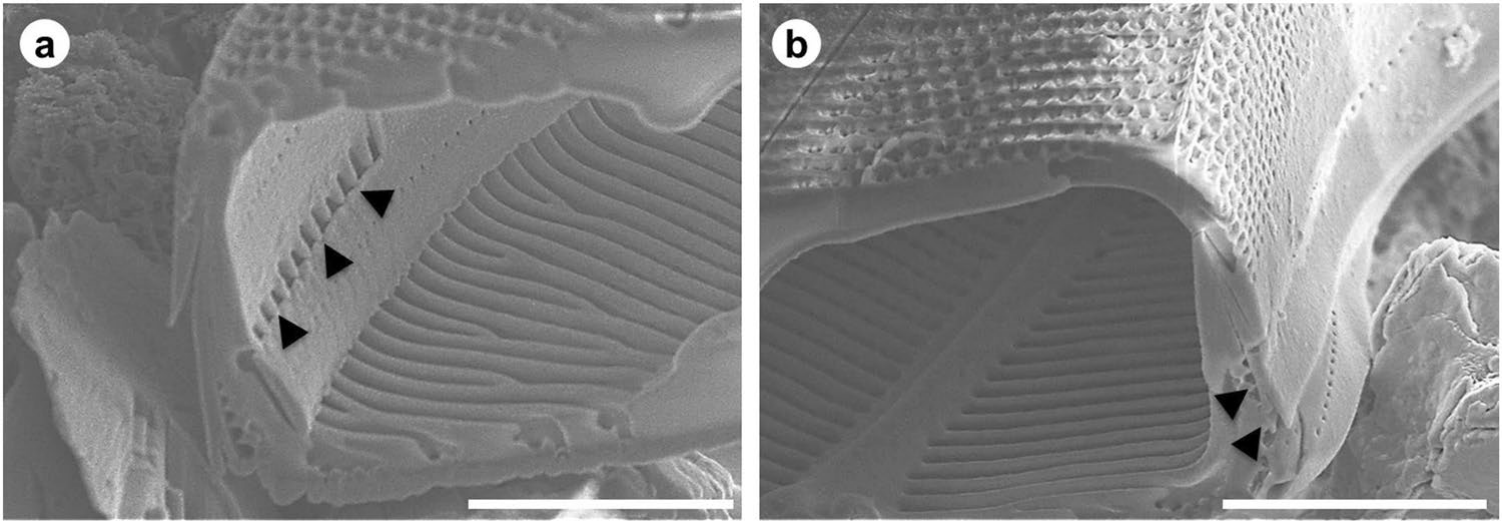
SEM image of an FIB cross-section through the *D. geminata* frustule, showing an overlapping epivalve and interlocking of adjacent girdle bands (marked with arrows): (**a**) left side, (**b**) right side. Scale bars: 10 µm.

**Figure 6:**
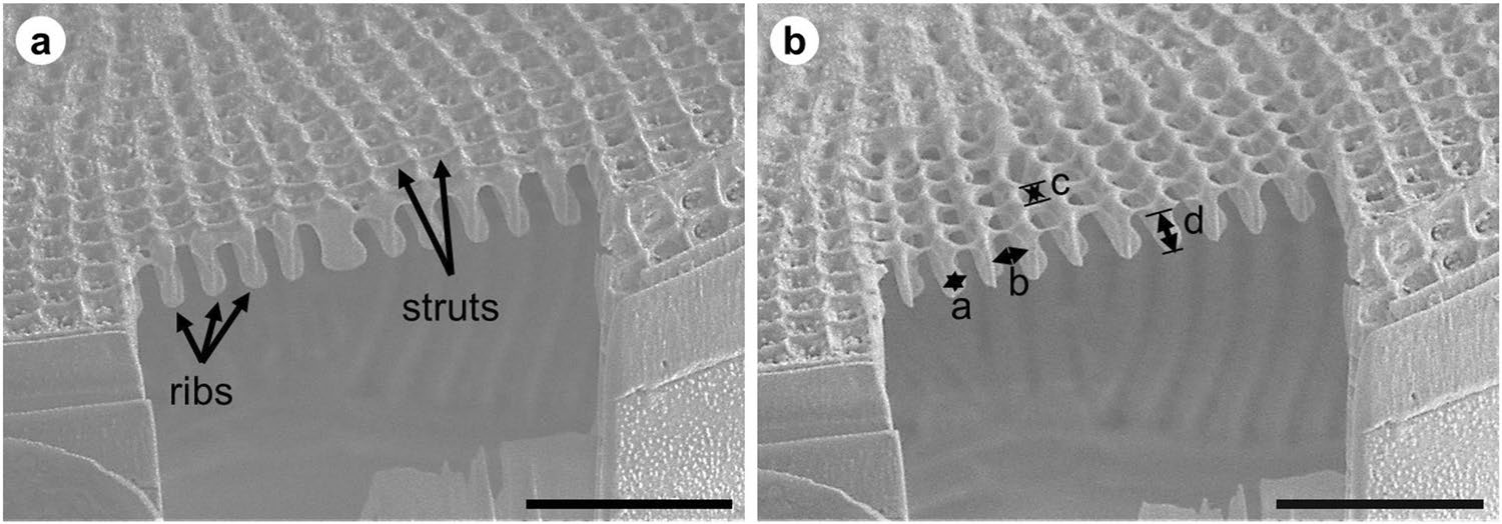
(**a**) SEM images of an FIB cross-section of the *D. geminata* frustule showing internal ribs. (**b**) The cross-section after further cuts with marked dimensions. Scale bars: 5 µm.

Images of the internal surface of the frustule, which were obtained using the FIB–SEM technique (Fig. 5), show that the major structural features are ribs that extend from the centre of the valve towards the girdle. These structures and the interior of the frustule are specifically depicted using nano-XCT (Figs 2b–d and 4). Note that a substantial portion of each rib is forked to compensate for the difference in the dimensions along the outer and inner edges. More details of the ribs are shown in Fig. 6.

The width (0.58 ± 0.03 µm), height (1.25 ± 0.15 µm) and the distances between ribs (1.05 ± 0.07 µm) and struts (0.49 ± 0.04 µm) of the rib sections were determined from SEM images of frustule cuts (see Table 3). The dimensions of the ribs of the frustules were also measured via nano-XCT (see Figs 2d and 4 (insets) and Table 3). The width of a single rib (a) is, on average, 0.5 µm; the distance between the ribs (c) is 1.1 µm; the depth (height) of the rib (b) is 1.1 µm; and the width and height of the strut are 0.5 µm and 0.3 µm, respectively.

**Table 3:** Average dimensions with standard deviations of the ribs of the *D. geminata* cell structure marked in Figs 2, 4 and 6.

Imaging method	Width [µm]	Height [µm]	Distance between ribs [µm]	Gap between struts [µm]
SEM	0.58 ± 0.03	1.25 ± 0.15	1.05 ± 0.07	0.49 ± 0.04
Nano-XCT	0.53 ± 0.10	1.10 ± 0.14	1.09 ± 0.01*	0.50 ± 0.09

**Figure 7:**
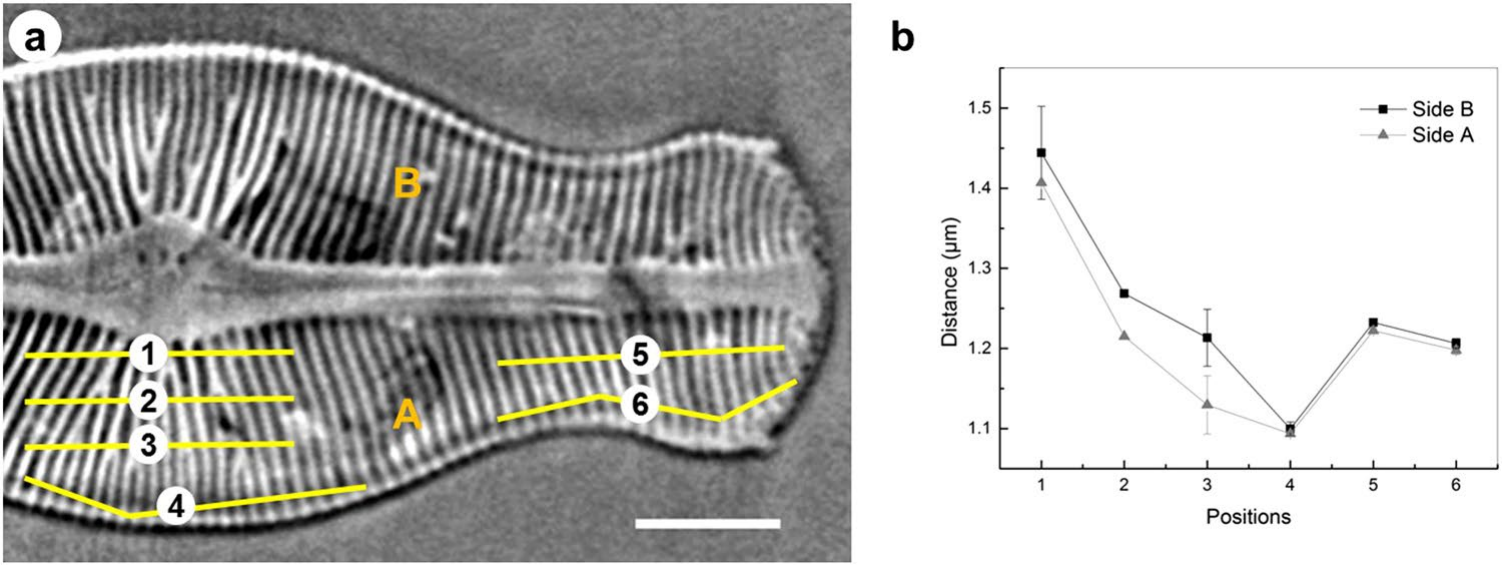
(**a**) The measurement of the rib distance at several positions (from 1 to 6) in the valve view of the *D. geminata* frustule. (**b**) Statistics of the distance between the ribs from centre to centre and from the centre area to the edge area of the *D. geminata* frustule. Scale bar: 10 µm.

*The distance between ribs is position 4 in Fig. 7.

To more specifically show the distance between the ribs and find the regular distribution law of the ribs, the distances between ribs at various positions in the valve view in the Z direction were calculated. Thus, positions 1, 2, 3, and 4 were chosen for the central region and positions 5 and 6 were chosen for the part of the foot pole (head pole). From the measurements of the distance between ribs at these positions at both sides, a curve was plotted, as shown in Fig. 7b. Positions 1–4 in Fig. 7b show a regular pattern in the central region, in which the distance of ribs decreased at a position far from the symmetry axis (apical axis). Additionally, the rib distance from positions 5 and 6 in the part of the head pole. follows the same rule. Because *D. geminata* is a pennate diatom and the ribs are symmetrical about the apical axis along the raphe slit, the other side B also follows this rule (Fig. 7b). Moreover, the width of the ribs in the central region are much larger than that in other regions, with a maximum rib width (Figs 2, 4 and 7) of approximately 1.4 µm. This observation is in agreement with our expectation that the rib’s distance in this region is larger than in other regions. The morphology of the ribs in the centre region is similar to that of the limb of a tree that extends to several branches (ribs).

The transapically oriented ribs are interconnected with short apically oriented struts, which contribute to the development of the areolae (pore-like) pattern on the external surface, which is much smoother than the internal one because of the architecture.

In summary, the entire 3D data set of the frustule that was determined using nano-XCT provides detailed morphological information, not only from the surface but also from the valve interior. The detailed 3D structure provides a complete nondestructive 3D view, which provides a basis for further modelling of the diatom structure.

## Conclusions

A detailed study of internal structures of *D. geminata* frustules using advanced high-resolution imaging is reported in this paper. The nano-XCT-measured 3D data (Tables 1 and 3) from a single frustule were determined at different positions. These data are not exactly the same as observations made using SEM, which were acquired from different frustules. The subtle differences between these measurements based on nano-XCT and SEM result from the variation in size of the diatom frustules, even in cases where the measured specimens represent the same species. Note that we did not use the identical frustule for imaging with both techniques.

Nano-XCT provides access to additional dimensions, e.g. the depth of the raphe (Fig. 2b) and depth of the stigma (Fig. 2c,d). Because the tomographic data set allows arbitrary cross-sections to be taken through any region of the frustule, measurement of, for example, the distance between the ribs at various locations (Fig. 7) becomes possible.

To summarize, nano-XCT provides a nondestructive high-resolution 3D data set of the diatom structure with detailed morphological information. This detailed 3D structural information from nano-XCT, which is based on arbitrary cross-sections through the diatom, provides novel information regarding the diatom substructure. The 3D data based on nano-XCT were validated via SEM images of FIB cross-sections through the ROI, and the dimensions of diatom substructures based on tomographic data were confirmed. The 3D information of the diatom substructures enables the validation and improvement of models related to mechanical parameters such as strength and stiffness.

## Experimental Materials and Methods

### Materials

The *D. geminata* studied in this paper was sampled in the Wisłoka River, Krempna Village, Subcarpathian Province in SE Poland. The expedition was conducted in July 2013. The following environmental conditions were measured during the sampling: pH = 7.9, conductivity = 276 µS cm^−1^, and water temperature = 21.0 °C. The water contained 2.95 mg L^−1^ of chlorides, 0.12 mg L^−1^ of nitrates, and 16.7 mg L^−1^ of sulphates. *D. geminata* samples were carefully scraped from boulders located inside the riverbed using a scrub brush with synthetic bristles. The fresh samples were immediately transported in special plastic boxes with riverine water to the laboratory (Warsaw University of Technology) for further study. In the laboratory, the cells were separated from the stalks. The scraped material was inserted into nylon-mesh filtration bags (Carl–Roth, GmbH, Germany) and immersed in distilled water for dialysis to remove dissolved salts from the natural stream waters. The dialysed material was then sonicated for 12 h using a CD-4860 digital ultrasonic cleaner (Xiejian, Guangdong, China) in continuous mode without heating. The cells collected from the tube were boiled in 37% hydrogen peroxide (H_2_O_2_) to remove organic matter. The final suspension was washed several times with distilled water and dried in a vacuum dryer at 37 °C for 12 h.

### Experimental techniques

#### Scanning electron microscopy of diatom frustules

The frustules of *D. geminata* from the water suspension were spread over a double-sided adhesive carbon tape on an aluminium pin disc using a pipette. For imaging at highest resolution via field-emission scanning electron microscopy (FE-SEM), samples were coated with Au/Pd (7 nm layer) using a high-vacuum sputter coater (Leica EM SCD500, Germany). Cross-sectioning and imaging were carried out with a dual-beam FIB–SEM tool (Hitachi NB5000, Japan) using acceleration voltages of 3.0 kV to 5.0 kV for the electrons.

#### Nano X–ray computed tomography (nano-XCT) of diatom frustules

A Nano-XCT tool (Xradia nano*-*XCT-100, US) was used in Zernike phase contrast imaging mode to image the frustules of *D. geminata* ^32^. The detailed experimental setup of the nano-XCT tool is described in Li *et al*. (2016)^33^. The isolated and dried frustule of *D. geminata* was mounted on the top of a needle and a gold fiducial marker was carefully positioned on top of the sample for the alignment of the individual images acquired at several tilt angles for tomographic reconstruction. The complete tomographic data set comprised 401 images, which were collected over 180° with an exposure time of 160 s for each individual image. These images were aligned using a custom plugin in ImageJ^34^ and subsequently reconstructed using the Xradia Inc. commercial software package^32^.
